# Chagas’ heart disease: gender differences in myocardial damage assessed by cardiovascular magnetic resonance

**DOI:** 10.1186/s12968-016-0307-5

**Published:** 2016-11-28

**Authors:** Antonildes N. Assunção, Michael Jerosch-Herold, Rodrigo L. Melo, Alejandra V. Mauricio, Liliane Rocha, Jorge A. Torreão, Fabio Fernandes, Barbara M. Ianni, Charles Mady, José A. F. Ramires, Roberto Kalil-Filho, Carlos E. Rochitte

**Affiliations:** 1 Heart Institute, InCor, University of Sao Paulo Medical School, Cardiovascular Magnetic Resonance and Computed Tomography Sector, Av. Dr. Enéas de Carvalho Aguiar, 44, Andar AB, Cerqueira César, São Paulo, SP 05403-000 Brazil; 2 Department of Radiology, Brigham and Women’s Hospital, Boston, MA USA

**Keywords:** Chagas’ heart disease, Gender differences, Myocardial fibrosis, Myocardial dysfunction

## Abstract

**Background:**

Since a male-related higher cardiovascular morbidity and mortality in patients with Chagas’ heart disease has been reported, we aimed to investigate gender differences in myocardial damage assessed by cardiovascular magnetic resonance (CMR).

**Methods and results:**

Retrospectively, 62 seropositive Chagas’ heart disease patients referred to CMR (1.5 T) and with low probability of having significant coronary artery disease were included in this analysis. Amongst both sexes, there was a strong negative correlation between LV ejection fraction and myocardial fibrosis (male *r* = 0.64, female *r* = 0.73, both *P* < 0.001), with males showing significantly greater myocardial fibrosis (*P* = 0.002) and lower LV ejection fraction (*P* < 0.001) than females. After adjustment for potential confounders, gender remained associated with myocardial dysfunction, and 53% of the effect was mediated by myocardial fibrosis (*P* for mediation = 0.004). Also, the transmural pattern was more prevalent among male patients (23.7 vs. 9.9%, *P* < 0.001) as well as the myocardial heterogeneity or gray zone (2.2 vs. 1.3 g, *P* = 0.003).

**Conclusions:**

We observed gender-related differences in myocardial damage assessed by CMR in patients with Chagas’ heart disease. As myocardial fibrosis and myocardial dysfunction are associated to cardiovascular outcomes, our findings might help to understand the poorer prognosis observed in males in Chagas’ disease.

## Background

Considered a neglected tropical disease, Chagas’ disease is caused by a protozoan parasite, *Trypanosoma cruzi* (*T. cruzi*) and has recently become a global health concern [1], due to immigration from endemic areas into the developed world [2, 3]. In Latin America, Chagas’ heart disease is still a major cause of heart failure despite a drop in incidence in the last decades [4].

Patients with Chagas’ heart disease have a poorer prognosis in comparison to other cardiomyopathies and, in particular, male gender has been associated to a higher mortality rates [5]. The reasons for this worse clinical prognosis remain unclear and, to our knowledge, there is a lack of studies about sex-related differences in myocardial damage in patients with Chagas’ heart disease.

Cardiovascular magnetic resonance (CMR) is able to assess the extent of the myocardial fibrosis (MF), which correlates to LV ejection fraction (LVEF) and is a marker of disease severity in Chagas’ heart disease [6, 7]. We sought to investigate whether the male gender is associated to greater myocardial damage assessed by CMR.

## Methods

From 2 previous studies [6, 8], 62 adults with chronic Chagas’ disease followed at Heart Institute (InCor), referred to CMR, from 2004 to 2012 and with low probability of having severe coronary artery disease (CAD) [9] were included in the study. We used a validated high-risk CAD score [9] based on the following clinical characteristics recorded at the time of CMR: age (−1 to 10 points), male sex (3 points), diabetes (2 points), hypertension (1 point), current smoking (2 points), hypercholesterolemia (1 point), family history of CAD (2 points), history of peripheral vascular disease (2 points), and chest pain symptoms (0–2 points). Patients who scored ≥18 points were excluded. Other exclusion criteria were previous myocardial infarction and severe valve disease. Patients in whom oral transmission from outbreaks of either beverage or food contaminations was suspected - which has unfortunately emerged in Brazil [10] - were not included in our study. The reason for this exclusion was the potentially more severe course of the disease following oral transmission [11]. The study was approved by the institutional review board for human subject studies, and all patients provided written informed consent.

### CMR

CMR was performed with a 1.5 T GE CV/i CMR System (Wakeusha Wisconsin). Ventricular function, volumes and mass were obtained from at least 10 short-axis ventricular slices, imaged with a steady state free precession pulse sequence, covering the entire left ventricle. Late gadolinium enhancement (LGE) images were acquired 10–20min after an intravenous bolus of 0.2 mmol/kg of gadolinium-based contrast, with an inversion-prepared gradient echo-sequence. The sequence parameters for cine/LGE imaging were, respectively: repetition time 3.9/7.1 ms, echo time 1.7/3.1 ms, flip angle 45°/20°, cardiac phases 20/1, views per segment 8/16 to 32, matrix 256 × 128/256 × 192, slice thickness 8/8 mm, gap between slices 2/2 mm and field of view 32 to 38/32 to 38 cm, inversion time none/150 to 250 ms, receiver bandwidth 125/31.25 kHz, number of excitations 1/1, acquisition every heart beat/every-other heart beat.

### Data analysis

All CMR images were analyzed with the CVI42 software (Circle Cardiovascular Imaging Inc. Calgary, Canada) by a trained observer. End-systolic, end-diastolic LV volumes, LV mass and LV ejection fraction were measured by standard methods [12]. For quantification of myocardial fibrosis (MF), we applied a semiautomatic thresholding technique to the LGE images with a signal intensity (SI) cutoff value of mean + 6 standard deviation (SD) (MF: SI ≥ meanSI of normal myocardium + 6 standard deviation of normal myocardium), which had best agreement with visual analysis. The location and extent of MF were assessed using the American Heart Association (AHA) segment model. Additionally, the pattern of LGE was classified as subendocardial, midwall, subepicardial or transmural. The myocardial heterogeneity or gray zone extent was defined as the myocardial region with pixels SI between meanSI + 6SD and meanSI + 7SD [13–15].

### Statistical analysis

Chi-squared or Fisher-exact tests and two-sample t-tests or Wilcoxon rank sum test for categorical or continuous data, respectively, were performed for comparing baseline characteristics across gender groups. The correlation between MF and LVEF was performed by Spearman’s test.

We assumed that MF lies on the causal pathway between gender and myocardial dysfunction in Chagas’ disease, as a mediator variable and we tested the following mediation assumptions [16]: gender has a significant effect on MF; gender has a significant effect on LVEF in the absence of the MF; MF has a significant unique effect on LVEF; and the effect of the gender on LVEF shrinks when the MF is added to the model. A Sobel-Goodman test was used to assess whether the amount of mediation effect was statistically significant [17]. After confirming the mediation assumptions, MF was added to the final model and a multivariate linear regression analysis was performed to determine the effect of gender on LVEF adjusted possible confounders. For all univariate and multivariate analysis, we used MF assessed as % LV mass since the normal population males have significantly greater myocardial mass [18].

All statistical analyses were performed with Stata 13.0 (StataCorp, Texas, USA) and a *P*-value < 0.05 was considered as statistically significant and all reported *P*-values were two-tailed.

## Results

### Clinical characteristics and ventricular remodeling

Patient characteristics are shown in Table 1. In comparison with 38 females (61.3%), 24 males (38.7%) had similar mean age (54 ± 11 vs. 55 ± 11, *P* = 0.55) as well as relative low prevalence of CAD risk factors. Although higher in males, both gender groups had low mean high-risk CAD score (4.3 ± 1.7 vs. 1.1 ± 1.1, *P* < 0.001). Moreover, 35 of our patients (56%) had either an invasive or coronary computed tomographic (CT) angiography during their follow-up and none of them had obstructive CAD.

**Table 1:** Characteristics of Patients with Chagas’ Heart Disease

Characteristics	Male (*n* = 24)	Female (*n* = 38)	*P*-value
Clinical Data			
Age – years*	54 (11)	55 (11)	0.55
Body mass index [kg/m^2^]^†^	26 (23–29)	25 (24–29)	0.55
Obesity - no. (%)	5 (21)	9 (24)	0.79
Hypercholesterolemia – no. (%)	9 (38)	16 (42)	0.72
Diabetes mellitus – no. (%)	4 (17)	2 (5)	0.19
Hypertension – no. (%)	8 (33)	14 (37)	0.78
Current smoker – no. (%)	4 (17)	2 (5)	0.19
CAD score^†^	4.3 (1.7)	1.1 (1.1)	**<0.001**
NYHA functional class > I - no. (%)	13 (54)	11 (29)	**0.04**
NYHA functional class^†^	2 (1–2.5)	1 (1–2)	**0.02**
GFR [mL/min per 1.73 m^2^]*	80 (26)	78 (26)	0.73
CMR			
LVEDVI [mL/m^2^]^†^	116 (97–161)	86 (72–108)	**<0.001**
LVESVI [mL/m^2^]^†^	74 (50–114)	34 (28–57)	**<0.001**
LV mass index [g/m^2^]^†^	74 (63–89)	51 (44–61)	**<0.001**
LVEF [%]^†^	37 (28–46)	58 (47–62)	**<0.001**
Aneurysm – no. (%)	7 (29)	6 (16)	0.21
RVESVI [mL/m^2^]^†^	80 (63–92)	68 (59–74)	**0.016**
RVEDVI [mL/m^2^]^†^	38 (32–50)	25 (22–34)	**<0.001**
RVEF [%]^†^	50 (36–59)	60 (52–65)	**0.005**
Myocardial Fibrosis – yes	21 (87)	27 (71)	0.21
Myocardial Fibrosis [grams]^†^	19 (9–30)	2.4 (0–12)	**<0.001**
Myocardial Fibrosis (% LV mass)^†^	12 (8–22)	3 (0–11)	**0.003**
Gray zone ^6SD-7SD^ [grams]^†^	2.2 (0–4.2)	1.3 (0–2.3)	**0.003**
LGE Patterns*	3.9 (0.9)	3.8 (1.0)	0.72

*LV* denotes left ventricular, *LVEF* left ventricular ejection fraction, *LVEDVI* left ventricular end-diastolic volume index, *LVESVI* left ventricular end-systolic volume index, *NYHA* New York Heart Association Functional Classification, *RVEF* right ventricular ejection fraction, *RVEDVI* right ventricular end-diastolic volume index, and *RVESVI* right ventricular end-systolic volume index, *LGE* late gadolinium enhancement, *GFR* Glomerular filtration rate. CAD score to assess the clinical probability of having high-risk CAD (low ≤7 points; intermediate, 8–17 points. Patients with high score, ≥18 points, were excluded from this study) [9]. *means (SD) and ^†^medians (25 and 75th centiles). Significant *P*-values (< 0.05) are shown in bold

Heart failure symptoms were significantly more frequent and severe among male patients (54% vs. 29% New York Heart Association [NYHA] class > 1, *P* = 0.04). Additionally, LV and RV remodeling was more pronounced in male than female patients, with males presenting significantly higher LV/RV dilation, LV mass and lower LV/RV ejection fraction than females.

### Myocardial fibrosis: presence, extent, distribution and patterns

**Fig. 1:**
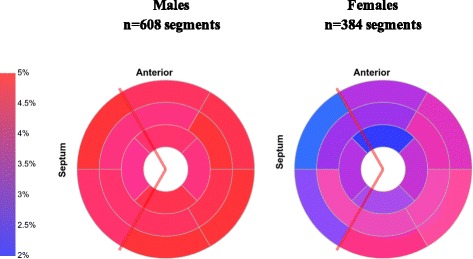
LGE frequency in male and female patients with Chagas’ heart disease. “n” illustrates the total of analyzed segments

Most of patients had MF (77%) and MF was similarly frequent in male and female patients (87% vs. 71%, *P* = 0.21) (Table 1). The distribution of MF in males and females was similarly frequent in the lateral and inferior LV segments, but significantly different in the septal (basal anterosseptal, *P* = 0.004) and apical segments (anterior and inferior apical, both *P* = 0.03) (Fig. 1). The amount of MF was significantly higher in male patients than in females (median 19.0 vs. 2.4 g, *P* < 0.001, and % LV mass 12 vs. 3%, *P* = 0.003) (Table 1).

**Fig. 2:**
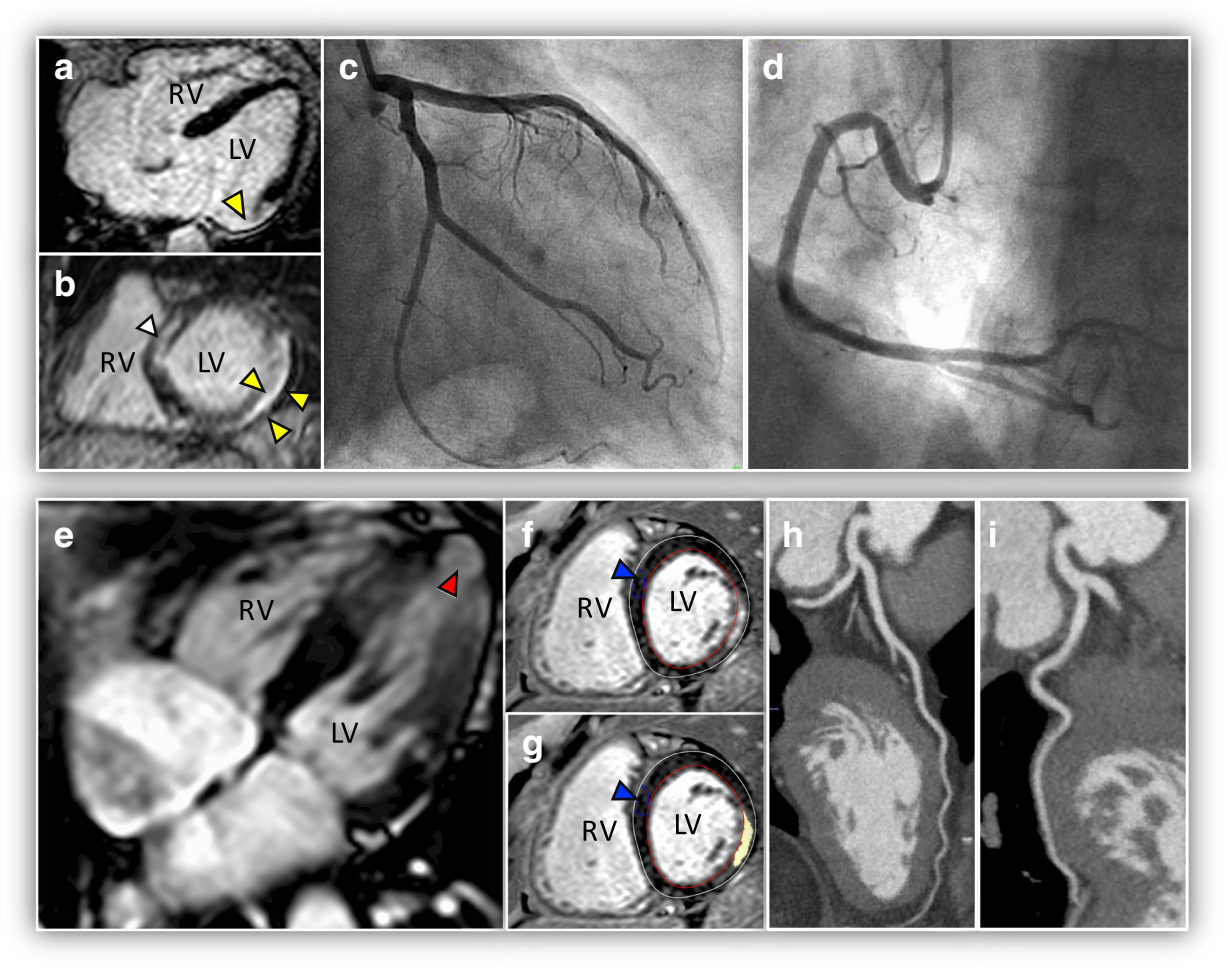
Examples of CMR, invasive and CT angiography in two representative patients with Chagas’ heart disease. *First patient (top row)*: **a** and **b** CMR reveals transmural LGE pattern in the LV lateral wall (*yellow arrow*) with concomitant septal midwall LGE (*white arrow*) and, by invasive angiography, (**c** and **d**) normal coronary arteries. *Second patient (bottom row):* (**e**) cine-CMR image reveals a classical Chagas’ heart disease finding, the vorticle aneurysm (*red arrow*), (**f** and **g**) LGE reveals subendocardial pattern in the lateral wall (*yellow arrow*) and, by CT angiography, (**h** and **i**) normal coronary arteries. Blue arrow (**f** and **g**) indicating the region of interest (ROI) for calculating the mean signal intensity (SI) of the reference normal myocardium

**Fig. 3:**
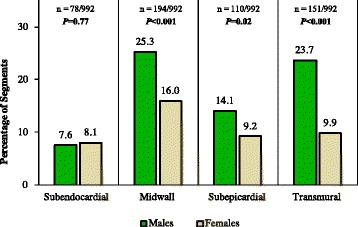
LGE patterns in male and female patients with Chagas’ heart disease. “n” illustrates the absolute frequency of the LGE pattern divided by the total of analyzed segments

Although LGE patterns indistinguishable from CAD were found in 23.1% of LV segments of patients (*n* = 151/992 transmural, *n* = 78/992 subendocardial), non-ischemic patterns were frequently and concomitantly observed (mean > 3 types of LGE patterns). Only 30% patients had subendocardial and/or transmural patterns and did not undergo any angiography during follow-up. Interestingly, these potentially ischemic patterns were also found in 24.6% of LV segments (*n* = 138/560) of those patients who had angiography (Fig. 2). Compared to females, males had more frequently transmural (23.6 vs. 9.9%, *P* < 0.001), subepicardial (14.1 vs. 9.2%, *P* = 0.02) and midwall patterns (23.8 vs. 15%, *P* < 0.001) (Fig. 3). Likewise, gray zone extent was higher in males (median 2.2 vs. 1.3 g, *P* = 0.003) (Table 1).

### Extent of myocardial fibrosis and LV remodeling

**Fig. 4:**
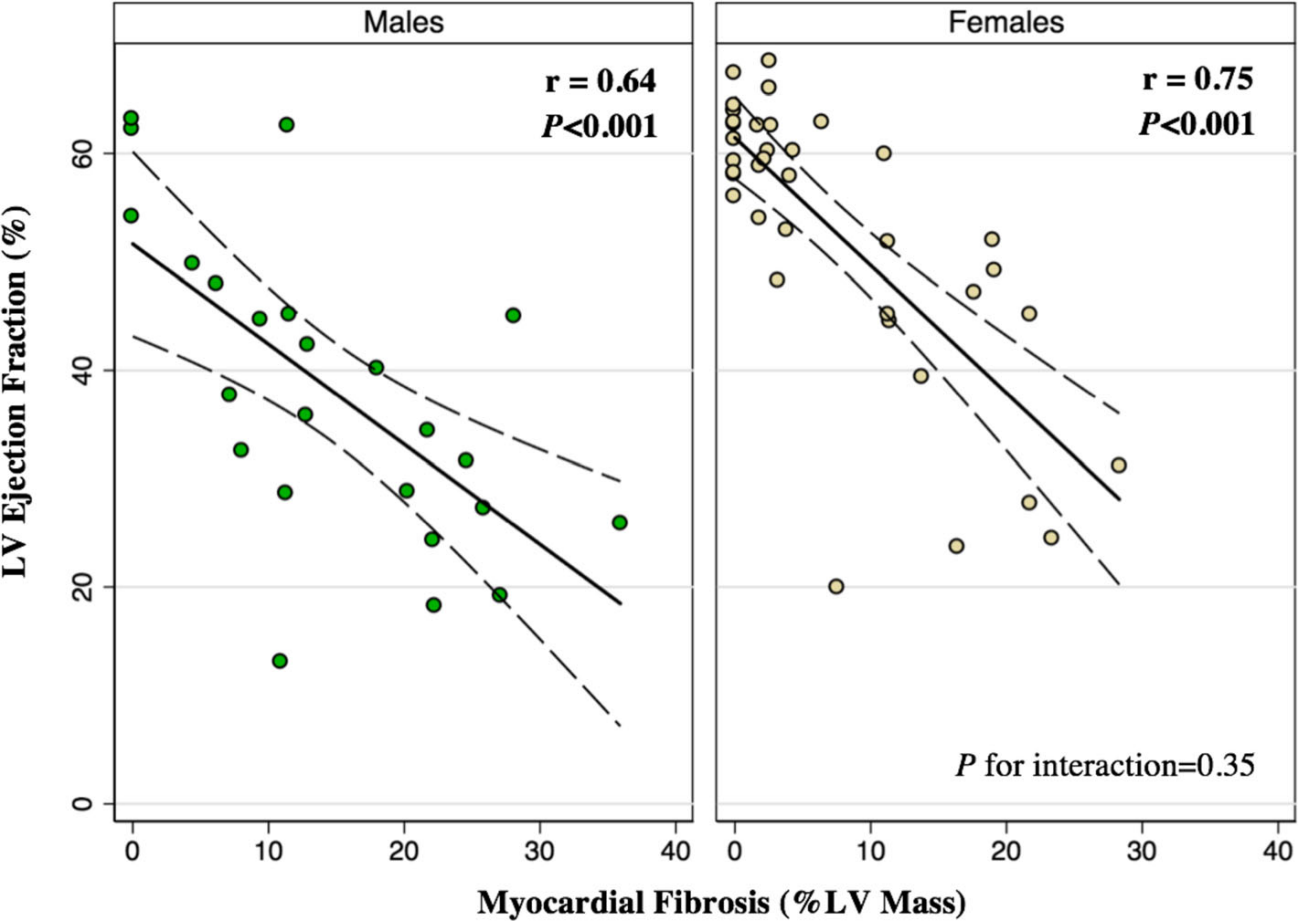
Sex-specific correlations between MF and LVEF

For all patients, MF (%LV Mass) was negatively correlated with LVEF (*r* = −0.75, *P* < 0.001) and this relationship was comparably strong among males (*r* = −0.64, *P* < 0.001) and females (*r* = −0.73, *P* < 0.001), with no indication of an effect modification by gender (*P*-value for interaction = 0.35) (Fig. 4).

**Fig. 5:**
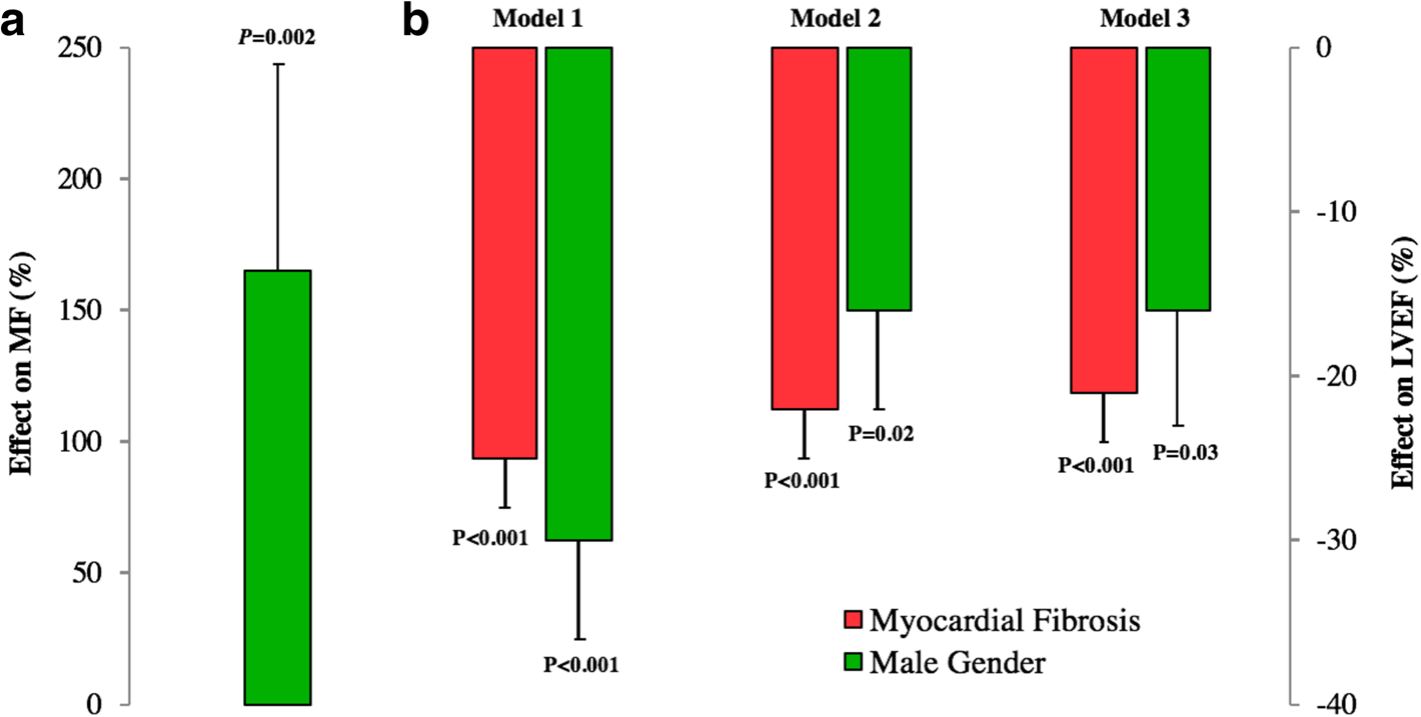
**a** Unadjusted effect of gender on MF and **b** effects of gender and MF (10-unit increase) on LVEF. Model 1 demonstrates unadjusted effects of gender and MF on LVEF. Model 2 demonstrates the effect of gender on LVEF when adjusted to MF. Model 3 demonstrates the Model 2 additionally adjusted for CAD risk factors (age, diabetes mellitus, hypertension, hypercholesterolemia, body mass index, active smoking). Natural logarithm transformation was used to improve normality and/or homoscedasticity of residuals. The effect was calculated from exponential linear regression coefficients (100 × [*e* ^*β*^ − 1]). LVEF was defined as left ventricular ejection fraction, and MF myocardial fibrosis (%LV Mass)

**Fig. 6:**
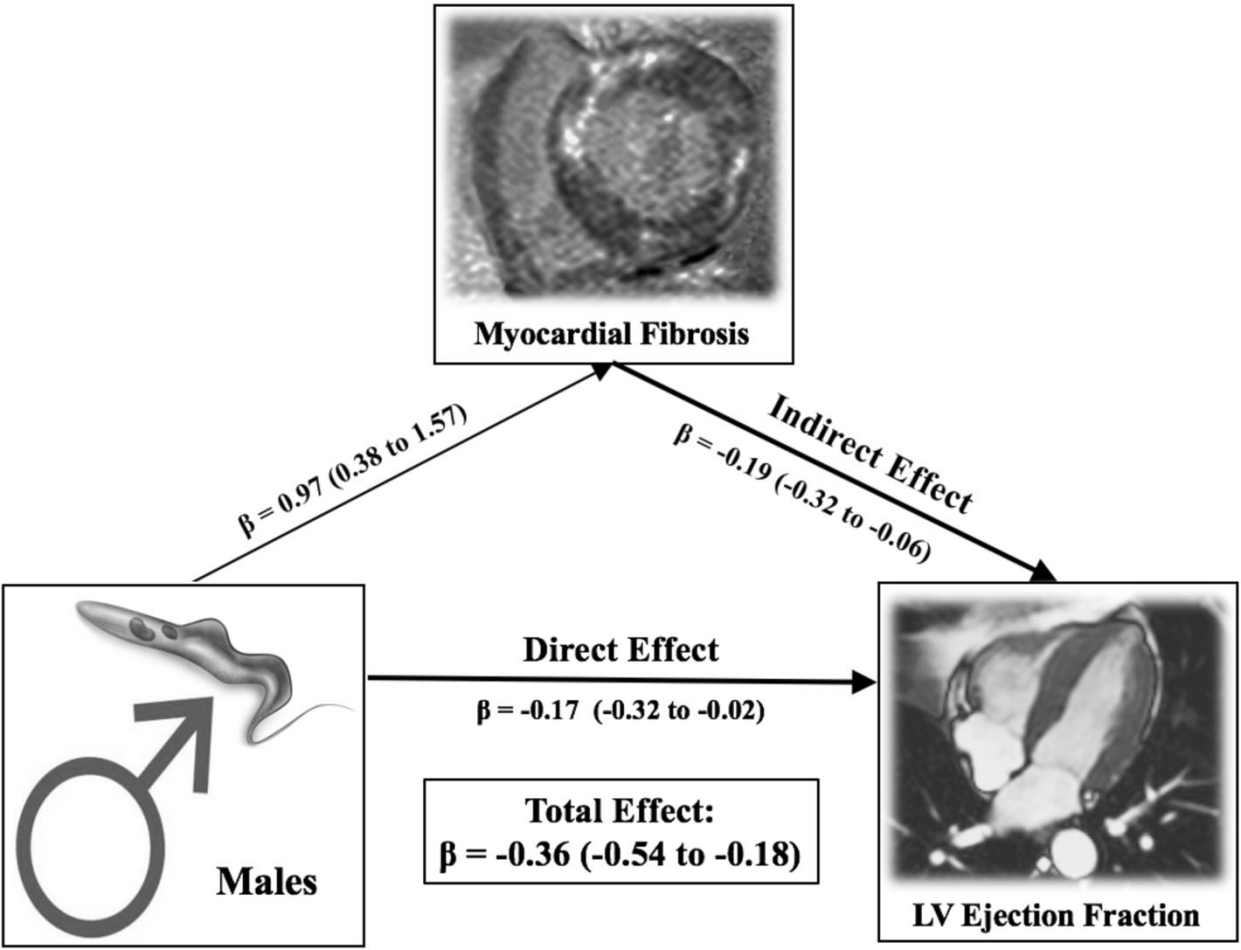
Mediation model illustrating indirect effect (through MF) and direct effect of gender on LVEF. β-coefficients of log-level linear regressions of Sobel-Goodman mediation tests. LVEF was defined as left ventricular ejection fraction, and MF myocardial fibrosis (%LV Mass)

Male gender was associated with 165% higher mean MF (95% confidence interval [CI] 11 to 320%) and 30% lower mean LVEF (95% CI 18–43%) compared to female (Fig. 5) in the unadjusted analysis. Although attenuated, the effect of gender on LVEF remained significant (*P* = 0.02) when MF was added as predictor in the model. In the mediation analysis (Figs. 5 and 6), approximately 53% of the total effect of gender on LVEF was mediated by MF (*P-*value for mediation analysis = 0.004). In a multivariate model to adjust for CAD risk factors, gender remained associated with myocardial dysfunction (Fig. 4).

## Discussion

In this study, we observed sex-related differences in myocardial damage assessed by CMR in patients with Chagas’ heart disease: males had significantly more adverse ventricular remodeling and greater MF, as well as different LGE patterns in comparison with females. Moreover, male gender was independently associated with reduced myocardial function and this effect was mostly mediated by MF. These findings might help to understand the observed gender-related differences in the pathogenesis of chronic Chagas’ heart disease.

Clinical data have revealed higher mortality among male patients with Chagas’ heart disease [5, 19], but this is the first study to demonstrate a significantly higher degree of myocardial damage in vivo. In our study, males patients had lower LVEF by 30% and higher MF by 165% on average compared to females. The pathogenesis of chronic Chagas’ heart disease is complex, but the inflammatory process with autoimmune reaction is considered the main pathway for myocardial damage, increasing MF, adverse left ventricular remodeling and heart failure severity [20–23]. In mouse models, this immune response against *T. cruzi* infection was more unfavorable in male species, and linked to gonadal hormone differences [24, 25]. MF measured by CMR is a key marker of myocardial damage in non-ischemic cardiomyopathies [26–28] and, likewise, in Chagas’ heart disease [6, 8]. For our patients, we found that the association between gender and LVEF was mediated by the amount of MF (53% of the effect was mediated). Hence, we hypothesized that male hormone differences might be an exposure to greater myocardial dysfunction in Chagas’ pathogenic, being the amount of MF a partly causal pathway.

Furthermore, the presence, size and heterogeneity of MF assessed by CMR have been described as independent predictors for death in ischemic and non-ischemic cardiomyopathies [29–31]. Besides the greater MF amount, we observed the transmural pattern of MF more frequently in males than females (23.7 vs. 9.9%, *P* < 0.001), as well as higher extent of the gray zones. Interestingly, the transmural pattern has been identified as independent predictor to ventricular tachycardia (4.1-fold greater) in Chagas’ heart disease [32]. A higher myocardial heterogeneity and/or larger gray zone extent have also been associated with a higher frequency of adverse cardiac events in non-ischemic cardiomyopathies [33].

There are several limitations in this study. First, although ventricular remodeling and MF have been linked to worse clinical prognosis, this study was not designed to investigate cardiac outcomes. Secondly, as this was a cross-sectional study in Chagas’ disease, the time of disease onset remained undetermined, and duration of exposure could influence myocardial damage. However, children aged <5 years are most likely to be infected in domiciliary vector transmission (by far, the most common mode of transmission in Brazil in the past) [1, 34], and the number of new cases of Chagas’ disease have dramatically been decreasing in Brazil since 1990 [1]. Consequently, our male and female patients, who were not different in age at the moment of CMR, may have similar exposure times. Thirdly, we recognize that as the patients have been followed at a tertiary hospital and current guidelines in Brazil do not recommend that patients with Chagas’ disease routinely undergo CMR, our results may not apply to other clinical settings and may suffer from some referral bias. Lastly, we did not assess the possible association between hormones and myocardial damage. Therefore our findings should be considered as hypothesis-generating for future studies adequately designed for addressing these issues.

## Conclusions

Our study demonstrated for first time gender-specific differences in myocardial damage in Chagas’ heart disease by CMR. Male gender was associated with a higher amount of myocardial fibrosis and worse ventricular remodeling. The relationship of these findings with clinical outcomes in Chagas’ heart disease warrants further investigation.

## Abbreviations

AHA: American Heart Association
CAD: Coronary artery disease
CMR: Cardiovascular magnetic resonance
GFR: Glomerular filtration rate
LGE: Late gadolinium enhancement
LV: Left ventricle
LVEDV: Left ventricular end-diastolic volume
LVEF: Left ventricular ejection fraction
LVESV: Left ventricular end-systolic volume
MF: Myocardial fibrosis
NYHA: New York Heart Association
RV: Right ventricle
RVEDV: Right ventricular end-diastolic volume
RVEF: Right ventricular ejection fraction
RVESV: Left ventricular end-systolic volume
SD: Standard deviation
SI: Signal intensity
